# Environmental, Institutional, and Demographic Predictors of Environmental Literacy among Middle School Children

**DOI:** 10.1371/journal.pone.0059519

**Published:** 2013-03-22

**Authors:** Kathryn T. Stevenson, M. Nils Peterson, Howard D. Bondell, Angela G. Mertig, Susan E. Moore

**Affiliations:** 1 Fisheries, Wildlife, and Conservation Biology Program, Department of Forestry & Environmental Resources, North Carolina State University, Raleigh, North Carolina, United States of America; 2 Department of Statistics, North Carolina State University, Raleigh, North Carolina, United States of America; 3 Department of Sociology & Anthropology, Middle Tennessee State University, Murfreesboro, Tennessee, United States of America; 4 Department of Forestry & Environmental Resources, North Carolina State University, Raleigh, North Carolina, United States of America; UC Davis School of Medicine, United States of America

## Abstract

Building environmental literacy (EL) in children and adolescents is critical to meeting current and emerging environmental challenges worldwide. Although environmental education (EE) efforts have begun to address this need, empirical research holistically evaluating drivers of EL is critical. This study begins to fill this gap with an examination of school-wide EE programs among middle schools in North Carolina, including the use of published EE curricula and time outdoors while controlling for teacher education level and experience, student attributes (age, gender, and ethnicity), and school attributes (socio-economic status, student-teacher ratio, and locale). Our sample included an EE group selected from schools with registered school-wide EE programs, and a control group randomly selected from NC middle schools that were not registered as EE schools. Students were given an EL survey at the beginning and end of the spring 2012 semester. Use of published EE curricula, time outdoors, and having teachers with advanced degrees and mid-level teaching experience (between 3 and 5 years) were positively related with EL whereas minority status (Hispanic and black) was negatively related with EL. Results suggest that school-wide EE programs were not associated with improved EL, but the use of published EE curricula paired with time outdoors represents a strategy that may improve all key components of student EL. Further, investments in teacher development and efforts to maintain enthusiasm for EE among teachers with more than 5 years of experience may help to boost student EL levels. Middle school represents a pivotal time for influencing EL, as improvement was slower among older students. Differences in EL levels based on gender suggest boys and girls may possess complementary skills sets when approaching environmental issues. Our findings suggest ethnicity related disparities in EL levels may be mitigated by time spent in nature, especially among black and Hispanic students.

## Introduction

Direct responses to global environmental crises can slow the tide of environmental degradation, but reversing the trend will require an environmentally literate citizenry. These direct responses have included converting nearly 12% of the earth’s land base to protected areas, which has undoubtedly altered the trajectory of species extinction [1]. Similarly, international climate change treaties guided by the UN Framework Convention on Climate Change (UNFCCC) and carbon markets represent the seeds of a response to global warming [2]. Comparable initiatives are associated with marine protected areas [3], water conservation and quality [4], and erosion mitigation [5]. The existence of these programs and development of future responses, however, depend entirely on publics who understand ecology, care about the environment, possess skills to assess environmental risk, and share a commitment to sustainability.

These four attributes of a citizenry capable of achieving sustainability are reflected by the four components of environmental literacy (EL): Knowledge, Affect, Cognitive Skills, and Behavior [6]. The environmental education (EE) movement revolves around promoting EL. Precursors to environmental education included both nature study and outdoor education early in the 19^th^ century, but environmental education as a field gained momentum in 1977 with the first intergovernmental conference on environmental education organized by the United Nations Education, Scientific, and Cultural Organization (UNESCO). The ensuing Tbilisi Declaration established EE objectives, and subsequent collaborations between environmental literacy experts have focused the definition of environmental literacy on the following topics: ecological knowledge, environmental attitudes and sensitivity, issue and action skills, and verbal and actual commitment to proenvironmental behavior [6], [7].

Environmental literacy helps to create a citizenry equipped to tackle current and emerging environmental concerns worldwide [8], and each component of EL is critical to this goal. Lack of empirical ecological knowledge about problems like extinction and climate change makes the problems insurmountable. For instance, a 2011 national poll found that 63% of adults in the United States “think there is solid evidence that the earth is warming,” which was down from 71% who saw solid evidence in 2008 [9]. If citizens do not accept overwhelming scientific evidence of warming trends, behavior to mitigate effects of global warming and support for government policies is highly unlikely [10]. Similarly, even an empirically aware public must care about the environment, have skills required to identify problems and solutions, and a willingness to act before the problems can be adequately addressed. That these components of EL are necessary to ensure pro-environmental behavior is both intuitive and supported by research. Hines, Hungerford, & Tomera [11] found that knowledge together with pro-environmental attitudes are requisites to environmentally responsible behavior, and a model has been further refined that includes in-depth knowledge about issues, personal investment in the environment, knowledge of and skill in using action strategies, and intention to act [12], all key components of EL [6].

Classroom activities have limited ability to change some components of EL, notably emotional connection to the environment (affect) and environmental behavior [13], and outdoor education has been promoted as a solution to this challenge. Time outdoors has been linked with the affective components of EL, a key predictor for proenvironmental behavior [14]. Duerdon and Witt [15] found that classroom activities were associated with improved environmental knowledge whereas field experiences were correlated with improved affect which indirectly improved behavior by activating knowledge. Skelly and Zajicek [16] also found time outdoors was a key predictor of pro-environmental attitudes in their evaluation of a gardening program. In Louisiana, horticulture teachers found that students participating in a program with an outdoor component were more aware of their role in the environment than students who did not participate in the program [17].

The dire need for EL has spurred legislation in several nations as well as attention at international conferences. In the United States, the Environmental Education Act of 1970 was one of the first major pieces of EE legislation and established the US Office of Environmental Education. Internationally, both the 1975 Belgrade Charter out of an International Workshop on Environmental Education hosted by the United Nations Environmental Program (UNEP) and Educational, Scientific and Cultural Organization (UNESCO), as well as the 1977 Tbilisi Declaration, brought international attention to the field of environmental education. Legislation stemming from these conferences included resolutions of the European Union Council of Environmental Education in 1988, the 1995 National Environment Statute in Uganda [18], and the Environmental Education Act of 1990 in the United States, which reestablished the Office of Environmental Education after its elimination in the 1980s [18]. Most recently, a groundswell of support for connecting children to nature spurred the No Child Left Inside Act of 2011 (NCLI) in the United States, which would have provided $100 million in funding for state EE efforts if passed. Though much of the aforementioned legislation supported specific programs, this act emphasized the role of outdoor education, integrated EE into formal schooling, and required the development of state EE standards, assessment, and teacher training through state-wide EL plans adopted by state boards of education [19].

Given the potential role of EL in addressing global environmental crises and the rapid expansion of EE around the world, there is surprisingly little empirical research addressing how EL is formed [20]. Further, even less research utilizes before-after, treatment-control designs. Blumstein and Saylan [21] suggest a “bunker mentality” within the environmental community may explain the reluctance to more formally evaluate the drivers of EL. Whatever the cause for limited evaluation, research addressing the drivers of EL is critical [22]. Although many studies examine factors contributing to at least one component of EL, [17], [23], [24], few if any address all four components or evaluate a broad suite of drivers. Efforts to standardize the way programs target and measure progress in all four elements include the National Environmental Literacy Assessment (NELA) project [7], [25] and the 2011 Framework for Environmental Literacy Assessment [6]. The NELA team developed the first major assessment tool for middle school students in the United States and the Framework project established guidelines for use in developing future assessment tools worldwide. Although these latest efforts have made progress in standardizing and measuring EL, no studies we are aware of have controlled for confounding factors including ethnicity, socio-economic background, school quality, and teacher training or addressed the degree to which specific EE efforts are effective in a school setting.

This study begins to fill the gap in EE research with an examination of participation in school-wide EE programs and how time spent in nature relates to EL in North Carolina, USA middle schools. We also accounted for use of published EE curricula; student attributes of gender, age, and ethnicity; teacher attributes of education level and years of experience; and school attributes of economic status, urban status, school type (charter or private vs. traditional public) and student-teacher ratio. We hypothesized that students’ EL scores would be: (1) positively related to participation in school-wide EE programs and use of published EE curricula, (2) positively related to class time spent in nature, (3) positively related to teacher development, measured by both having an advanced degree and years of teaching experience, (4) negatively related to student attributes historically associated with low academic performance (e.g., interest and performance in science wanes with student age [26], girls score lower than boys in science [27], and minority students score lower than white students in other academic areas [28]), and (5) negatively related to student-teacher ratios and enrollment in lower-income schools.

## Materials and Methods

### Ethics Statement

The North Carolina State University institutional review board (IRB # 2212) approved this study. All participants provided written informed consent. Students and their parents/guardians were given either a Passive Consent form or an Active Consent form, per the preference of the teacher and/or school. The Passive Consent form was only signed and returned if the parents/students did not want to participate. The Active Consent form was signed and returned to indicate consent to participate in the study.

### Sample Selection

We targeted 6^th^ and 8^th^ grade students because middle school students are developing cognitive abilities linked to the goals of EL and represent the latest and prime stage for influencing how students engage in society as citizens and environmental decision makers [7]. Additionally, we chose 6^th^ and 8^th^ graders so we could compare our results with the only other large scale assessment of EL available at the time that our study was conducted – Phase 1 of the National Environmental Literacy Assessment Project [7].

Sampling occurred in three stages: schools, teachers, and classrooms. We followed the three stage sampling model for two groups – an EE group randomly selected from schools registered with the NC Office of Environmental Education as having school-wide EE programs, and a control group randomly selected from 6^th^ and 8^th^ grade science classes in North Carolina that were not registered with the Office of Environmental Education. We generated the first stage of the EE sample from a list of all schools registered with the NC Office of EE (n = 40). We generated the first stage of the control sample from a list of all 665 middle schools in North Carolina. We omitted the 40 schools already included in the EE group and then randomly selected 40 schools for the control group from the remaining 625 schools.

From the two lists of 40 schools, we generated a list of 6^th^ and 8^th^ grade science teachers at each school. This process resulted in 135 teachers in the control group and 95 teachers in the EE group. We randomly selected 85 teachers from each group to recruit for the study and e-mailed each a letter of introduction and a brief recruitment survey. We sent up to four survey reminders to each teacher in 4–7 day increments. Of the 170 teachers contacted, 59 (34.7%) responded, and of these, 21 declined to participate and 38 consented, representing 20 members of the control group and 18 members of the EE group. Two teachers from each group later withdrew from the study. The 64% compliance rate among teachers who we successfully contacted could allow for bias if participating teachers were more environmentally oriented than other teachers. Because evaluating drivers of EL was more important than extrapolating findings to general assessments of EL in NC, this potential bias should not be problematic. Further, we measured teachers’ environmental orientations using the New Ecological Paradigm scale [29] and their scores were not related to student scores on the MSELS (r = −0.0186, p = 0.614), so if participating teachers were more environmentally oriented than others it should not bias student scores. Entering the pretest, we had 18 teachers participating from the control group (ten 6^th^ grade and eight 8^th^ grade teachers) and 16 teachers participating in the EE group (four 6^th^ grade teachers and twelve 8^th^ grade teachers). Though we contacted equal numbers of teachers in the control and EE groups and in 6^th^ and 8^th^ grades, differential teacher response rates prevented equal classroom numbers. If a teacher taught more than one 6^th^ or 8^th^ grade class, we asked them to flip a coin to randomly select one class for participation.

### Survey Instrument

All students in the study were given the Middle School Environmental Literacy Survey (MSELS) developed by the NELA team [7], [25]. The MSELS consists of eight sections that contribute to an overall environmental literacy score (Table 1). The MSELS was based on four instruments that reported established high validity and reliability – the Middle School Environmental Literacy Instrument [30], Children’s Environmental Attitude and Knowledge Scale [31], the Secondary School Environmental Literacy Assessment Instrument [32], and the Ecology Attitude Inventory [33]. In the 2008 national survey using the MSELS, the Chronbach alpha coefficients for each component of the MSELS fell between 0.701 and 0.869 with the exception of a three-item Issue Identification scale with an alpha coefficient of 0.389. The total reliability for the MSELS in this 2008 study was 0.82 [7]. Teachers were given a separate survey that asked about their use of EE, the degree to which they take their students outside, and their own training. Key questions included whether they use a published EE curriculum (e.g. Project WET, Project WILD, Project Learning Tree), if they visit natural areas during class time, and their experience in education and EE.

**Table 1:** Summary of MSELS contents and average pretest scores [7].

Environmental LiteracyConcept	Specific Conceptual Variables	Sample question	# of Items	MaximumScore	Averagepretest score	Averagepretest score %
**Ecological Knowledge** **(**Multiple choice**)**	Ecological Knowledge	If there were no decomposers lefton Earth, what would happen?	17	60	44.0	73.3%
**Environmental Affect and** **Awareness**(5-point Likert Scale)	Verbal Commitment (intention)	To save water, I would be willing touse less water when I bathe.	12	30	23.3	80.0%
	Environmental Sensitivity	To what extent do you spendtime outdoors alone?	11	25	18.8	
	Environmental Feeling	I love the environment.	2	5	4.0	
**Cognitive Skills** **(**Multiple choice**)**	Issue Identification	These three sections involve reading a passage, identifyingthe issues at hand, analyzing whatfactors are at play, andplanning a course of action.	3	20	8.0	41.0%
	Issue Analysis		6	20	10.5	
	Action Planning		1	20	8.2	
**Behavior**(5-point Likert Scale)	Actual Commitment	I do not separate things at home for recycling.	12	60	47.0	78.3%
**Total Score**				**240**	**163.6**	**68.2%**

The MSELS was organized into eight specific concept variables that were grouped into four environmental literacy concept component scores. Average pretest scores for the total sample are shown in raw score and percentage of maximum score for each component.

### Data Collection

In January of 2012, we visited all 34 classrooms. We administered the MSELS to the students and asked each teacher to complete the teacher survey. From April 11 to June 6, 2012, we returned to the same classrooms and administered the post-test (using the MSELS again) to the students. While visiting each classroom for the post-test, we also asked teachers to complete a shorter follow-up survey that asked about the time they spent outdoors with the participating class, their use of EE, and any further EE training they had received since the pretest.

We surveyed 856 students during the pretest and 846 during the post-test. We eliminated students who were absent during either the pretest or the post-test from the pre/post-test comparison yielding 739 students in the comparison. Use of published EE curricula and time spent outdoors were reported by the teachers in the teacher surveys at the time of the pretest and confirmed by the follow-up survey at the time of the post-test. Teacher attributes of higher education and years teaching experience were self-reported as questions in the teacher survey. Student attributes of age, gender, and ethnicity were self-reported as questions within the MSELS. For school attributes of locale, school-level socio-economic status (SES) measured by Title I status, student/teacher ratios, and charter or private school designation, we used data from the National Center for Education Statistics. The Title I program is authorized by the Elementary and Secondary Schools Act to give additional funding to schools with high percentages of low-income students [34], and Title I status can be used as a measure of school-level SES. Locale includes 12 categories: Large city, midsize city, small city, large suburb, midsize suburb, small suburb, fringe town, distant town, remote town, fringe rural, distant rural, and remote rural areas [35]. We collapsed these categories into urban (including all size cities and suburbs) and rural (including all size towns and rural areas).

### Data Analysis

We analyzed data using STATA software, version 12.1. Multiple linear regression analysis was used to model component scores for the pretest and the difference between the post-test and pretest as a function of membership in the EE group, spending time outdoors as reported by the teacher at the end of the study, use of published EE curriculum, teacher education level (Master’s degree or higher), years teaching experience, student attributes (age, gender, ethnicity) and school attributes (student/teacher ratio and Title I program). We also controlled for school locale and type of school (private or charter vs. traditional public) in all models. To account for the fact that students within the same classroom also are exposed to the same teacher and school attributes, we included a random effect for class. This approach captures the likelihood that students within the same classroom may have similar EL levels as opposed to independent random deviations of student scores. Additionally, we calculated robust standard errors to account for the possibility of unequal variances between individual students [36].

For each analysis (pretest and difference in scores), we modeled each component of the MSELS (Knowledge, Affect, Cognitive Skills, and Behavior) as well as Overall EL using the aforementioned independent variables. We calculated each of these scores according to the guidelines listed in the Phase One NELA study [7]. This method weighted the four components in the MSELS to contribute equally to the Overall EL score. When modeling the change in MSELS scores, we also included the pretest score as a predictor to control for the fact that students scoring high in the pretest had less potential for improvement in the post-test.

Modeling both pretest scores and modeling how scores changed between the pretest and post-test was necessary because pretest scores reflected EL levels when students entered the study in January, half way through the school year. Spring testing was conducted to facilitate comparisons with other studies using the MSELS [7], [25]. This meant that students had already been in their respective classes for several months – with the same teacher, often employing the same curricular strategies including use of environmental and outdoor education. Some variables may predict pretest scores and not change in EL, because the impact of that particular variable was exerted during first semester of the school year. Thus the pretest models allow assessment of variables with relatively rapid impacts whereas the change model allows assessment of the variables that may not have exerted a discernible effect during the first semester.

## Results

### Descriptive Statistics

There were 415 students in the control group (240 6^th^ graders and 175 8^th^ graders) and 324 students in the EE group (70 6^th^ graders and 254 8^th^ graders). Almost half (49.1%) of the students in the study spent some class time outdoors and 42.1% were exposed to published EE curricula. The average student age was 12.7 years, and there were slightly more females (53.1%) than males. The students were primarily white (70.6%) and black (14.6%), with smaller percentages of Hispanic students (8.2%), Asian students (3.4%) and Native American/Alaskan students (3.3%). Half (50.0%) of the teachers in the study held Masters degrees, and the average experience level was 9.2 years teaching. Over half of the students were from schools with Title I programs (56.3%), and from rural communities (66.0%). The average school-wide student-teacher ratio was 14.6, and 26.8% of the students in the study were enrolled in Charter or private schools. Average pretest Cognitive Skills were considerably lower than the other three components measured in the MSELS (Table 1).

### EE and Outdoor Education

Our results partially support hypothesis 1 because attendance at EE schools often had either a non-significant or negative relationship with EL, but classroom engagement in published EE curriculum was positively related to EL. Students in the EE schools pretested lower in Affect and Behavior (Table 2) and changed in the same ways as the control group over the course of the semester (Table 3). Further, membership in the EE schools was the most important negative predictor of pretest scores for the Affect component (Table 2). The explanatory power of the Affect and Behavior pretest models was low (R^2^ = 0.071 and 0.054, respectively), suggesting that although there was a negative relationship between school-wide EE programs and Affect and Behavior scores, important drivers of Affect and Behavior (e.g., behavioral norms, values [37], parental attributes, presence of a role model for environmental stewardship [38]) were not accounted for in the models. Although all schools in the EE group were registered with the NC Office of Environmental Education as having EE programs, only four of the teachers in this group indicated that their students participated in a formal EE program. Similarly, none of the teachers in the control group were associated with schools registered with the Office of EE, but three reported their students participated in a formal EE program.

**Table 2:** Pretest MSELS Scores.

	Knowledge^a^		Affect		Cognitive Skills^a^		Behavior		Overall MSELS Score^a^	
	Beta	*p*	Beta	*p*	Beta	*p*	Beta	*p*	Beta	*p*
EE Group^b^	1.871	0.058	−1.782***	<0.001	4.497	0.079	−1.328**	0.004	3.092	0.309
Use of Published EE Curriculum^c^	0.647	0.540	0.281	0.419	4.095	0.055	−0.123	0.752	4.801*	0.045
Time in Natural Areas^d^	2.366*	0.020	1.188***	<0.001	2.65	0.090	0.725*	0.035	6.935**	0.001
Teacher Has Masters^e^	5.259***	<0.001	1.054**	0.010	2.469	0.224	1.362***	<0.001	10.248***	<0.001
Years Teaching^f^										
* 3–5 Years*	3.320	0.200	0.345	0.656	9.685***	<0.001	−0.522	0.32	11.995**	0.004
* 6–8 years*	0.199	0.911	0.001	0.998	3.418	0.122	−1.980***	<0.001	1.081	0.732
* 9–11 years*	−0.809	0.683	−0.635	0.375	5.05	0.155	−1.602**	0.004	1.362	0.780
* 12 or more years*	0.278	0.869	0.813	0.229	5.441	0.145	−1.870**	0.005	4.505	0.248
Student Age (in years)	−0.483	0.477	−0.150	0.414	−0.344	0.645	0.249	0.270	−0.272	0.845
Student Gender (Female) g	−1.936**	0.001	1.234**	0.001	2.208**	0.009	0.391	0.360	1.766	0.151
Student Ethnicity^h^										
* American Indian/Alaskan Native*	−7.311***	<0.001	0.426	0.661	−5.683*	0.018	1.812	0.165	−11.041**	0.001
* Asian*	2.132	0.147	1.081	0.155	1.969	0.493	0.495	0.686	5.986	0.108
* Hispanic*	−5.611***	<0.001	0.115	0.892	−4.179*	0.044	−0.264	0.822	−10.183**	0.001
* Black*	−5.307***	<0.001	0.018	0.970	−5.865**	0.003	0.500	0.384	−10.441***	0.001
Student/Teacher Ratio^i^	−0.561*	0.020	0.007	0.947	−0.336	0.491	0.166	0.066	−0.686	0.285
Charter or Private School^j^	0.482	0.712	0.449	0.263	−0.268	0.928	1.362**	0.002	1.837	0.565
Title I Program^k^	−1.388	0.100	−0.626	0.152	0.401	0.838	−1.958***	0	−3.823	0.149
Urban^l^	0.703	0.452	−0.111	0.754	3.31	0.109	0.332	0.387	4.338	0.119
Intercept	50.692***	<0.001	45.213***	<0.001	20.480**	0.004	45.294***	<0.001	160.509***	<0.001
R^2^_a_	0.219		0.071		0.168		0.045		0.217	
N	731		731		731		731		731	
rho	0.045		0.01		0.124		0		0.078	

a Random effect is significant (non-zero), and rho is the proportion of residual variance explained by the within classroom effect.

b EE group membership (No = 0, Yes = 1).

c Used published environmental education curriculum (No = 0, Yes = 1).

d Spent time in outdoors during class time (No = 0, Yes = 1).

e Teacher holds Master’s degree (No = 0, Yes = 1).

f Reference group is teachers with 0–2 years teaching experience (0–2 years = 0, 3–5 years = 1, 6–8 years = 2, 9–11 years = 3, 12 or more years = 4).

g Student gender (Male = 0, Female = 1).

h Reference group for student ethnicity is white students.

i School-wide average student/teacher ratio.

j School is either a charter school or private school (No = 0, Yes = 1).

k School has a Title I program (No = 0, Yes = 1).

l School categorized as urban (No = 0, Yes = 1).

Each labeled column represents a separate multiple regression model for each section of the MSELS as well as total scores. Metric coefficients and p-values are displayed. Each model includes random effects for classroom and all standard errors are robust.

* p<.05

** p<.01

*** p<.001.

**Table 3:** Difference in MSELS Scores between pretest and posttest.

	Knowledge^a^		Affect		Cognitive Skills^a^		Behavior		Overall MSELS Score^a^	
	Beta	*p*	Beta	*p*	Beta	*p*	Beta	*p*	Beta	*p*
EE Group^b^	1.594	0.278	−0.114	0.711	−0.245	0.858	−0.364	0.415	0.257	0.917
Use of Published EECurriculum^c^	1.316	0.195	0.021	0.911	3.549**	0.004	0.273	0.516	4.349*	0.024
Time in Natural Areas^d^	1.64	0.18	0.041	0.858	0.621	0.591	1.292**	0.001	3.235	0.13
Teacher Has Masters^e^	1.525	0.289	0.119	0.636	2.1	0.117	1.428***	0.001	4.06	0.109
Years Teaching^f^	0	.	0	.	0	.	0	.	0	.
* 3–5 Years*	1.873	0.476	0.123	0.569	−0.083	0.972	2.027	0.098	2.345	0.676
* 6–8 years*	−1.143	0.665	−0.624**	0.003	0.468	0.837	0.391	0.747	−0.708	0.901
* 9–11 years*	−1.714	0.515	−0.681	0.062	0.307	0.897	0.571	0.587	−1.389	0.794
* 12 or more years*	−0.798	0.75	−0.097	0.714	0.713	0.763	0.35	0.734	−0.101	0.984
Student Age (years)	−0.712	0.218	−0.032	0.781	−0.585	0.219	−0.614*	0.034	−2.027*	0.032
Student Gender (Female) g	1.377*	0.019	0.062	0.745	0.705	0.418	0.087	0.813	1.701	0.211
Student Ethnicity^h^										
* American Indian/Alaskan* *Native*	−0.151	0.947	0.203	0.641	−5.572**	0.002	−0.71	0.576	−6.443	0.137
* Asian*	0.405	0.746	<0.001	0.999	−0.296	0.918	0.378	0.66	−0.259	0.941
* Hispanic*	−2.125	0.125	−0.282	0.624	2.415	0.148	−0.565	0.5	0.44	0.885
* Black*	−4.341***	<0.001	0.042	0.890	−3.699**	0.002	0.274	0.651	−7.146***	<0.001
Student/Teacher Ratio^i^	−0.096	0.744	0.082	0.117	−0.355	0.289	−0.072	0.606	−0.382	0.532
Charter or Private School^j^	1.37	0.329	−0.151	0.543	2.589	0.108	0.451	0.44	3.723	0.166
Title I Program^k^	−1.608	0.142	0.05	0.819	0.225	0.882	−0.657	0.122	−1.773	0.435
Urban^l^	−0.848	0.513	0.274	0.109	−2.966*	0.046	0.153	0.741	−4.442	0.064
Pretest Score^m^	−0.373***	<0.001	−0.205***	<0.001	−0.490***	<0.001	−0.589***	<0.001	−0.311***	<0.001
Intercept	18.426***	<0.001	8.457***	<0.001	17.895***	<0.001	27.848***	<0.001	58.447***	<0.001
R^2^_a_	0.156		0.145		0.269		0.308		0.153	
N	731		731		731		731		731	
rho	0.065		0		0.087		0.034		0.041	

a Random effect is significant (non-zero), and rho is the proportion of residual variance explained by the within classroom effect.

b EE group membership (No = 0, Yes = 1).

c Used published environmental education curriculum (No = 0, Yes = 1).

d Spent time in outdoors during class time (No = 0, Yes = 1).

e Teacher holds Master’s degree (No = 0, Yes = 1).

f Reference group is teachers with 0–2 years teaching experience (0–2 years = 0, 3–5 years = 1, 6–8 years = 2, 9–11 years = 3, 12 or more years = 4).

g Student gender (Male = 0, Female = 1).

h Reference group for student ethnicity is white students.

i School-wide average student/teacher ratio.

j School is either a charter school or private school (No = 0, Yes = 1).

k School has a Title I program (No = 0, Yes = 1).

l School categorized as urban (No = 0, Yes = 1).

m Score on pretest.

Each labeled column represents a separate multiple regression model for each section of the MSELS as well as total scores. Metric coefficients and p-values are displayed. Each model includes random effects for classroom and all standard errors are robust.

* p<.05.

** p<.01.

** p<.001.

**Figure 1:**
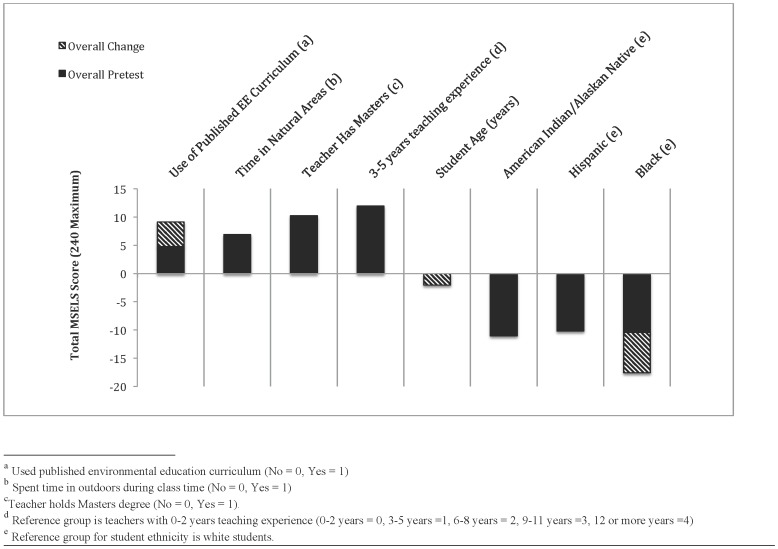
Significant predictors of pretest and change in EL. Variables present represent the significant predictors of pretest and/or change in Overall environmental literacy from the full models represented in Table 2 and Table 3. Pretest scores represent total MSELS score at time of pretest and change in scores represents difference between pretest and post-test scores attributed to each variable independent of the others in the model.

Students exposed to published EE curricula improved more than students who were not, especially with respect to Cognitive Skills. Four teachers in the control group and six teachers in the EE group reported use of a published EE curriculum. The most frequently used EE curriculum was Project WILD (29.1% of students exposed) followed by Project WET (28.2%) and Project Learning Tree (12.8%), although most teachers reported to using multiple curricula and at least 13 curricula were listed. Students whose teachers used at least one published EE curriculum entered the study pretesting higher on Overall EL (Table 2). Students engaged in a published EE curriculum also improved more than other students in the Cognitive Skills component and Overall (Table 3, Figure 1). The use of published EE curricula was the only positive predictor of the change in the Cognitive Skills component measured in this study (Table 3).

Both the pretest and change in EL models support hypothesis 2 by suggesting that taking students outside was positively related to EL. Spending time in nature was positively related to the pretest scores for all components except Cognitive Skills (p = 0.09) and was the most important factor predicting the Affect pretest scores in terms of significance and magnitude (Table 2). Time outdoors was the most important positive predictor of change in the Behavior component along with whether the teacher had a Master’s degree (Table 3).

### Teacher Attributes

Our results support hypothesis 3 by suggesting teacher development, primarily through advanced degrees, is associated with improved EL among students. Teachers with Master’s degrees had students who pretested higher in all components of EL except for the Cognitive Skills component (Table 2), and those same students improved more than others in the Behavior component (Table 3). Of the teachers that had Master’s degrees, most (75%) held a degree in education or science education. Advanced degrees for teachers had the most important positive relationship with the pretest of Knowledge in terms of magnitude and significance (Table 2). The relationships between student EL and teacher experience were mixed. Teachers with 3–5 years of teaching experience had students that outperformed those with fewer than 3 years of teaching experience in the pretest of Cognitive Skills and Overall EL (Table 2, Figure 1). Teachers with 6 or more years of experience had students that pretested lower in Behavior than teachers with fewer than three years of experience (Table 2). Students with teachers of 6–8 years teaching experience also did not improve as much in Affect as those with teachers with fewer than 3 years of experience.

### Student Attributes

Our results support hypothesis 4 in terms of ethnicity and age but are mixed with regards to student gender. Girls pretested lower than boys in Knowledge but higher in Affect and Cognitive Skills (Table 2), although they improved more than boys in Knowledge (Table 3). Older students performed the same as younger students in the pretest (Table 2) and improved less than younger ones in Behavior and Overall scores (Table 3, Figure 1). Minority students fell behind in several components of EL. Native American students pretested lower than white students in the Knowledge and Cognitive Skills components as well as Overall (Table 2, Figure 1) and improved more slowly than white students in Cognitive Skills during the semester (Table 3). Hispanic students followed the same pretest patterns as Native American students (Table 2, Figure 1), but improved as much as white students over the course of this study (Table 3). Black students entered the study behind white students in the same areas as Native American and Hispanic students (Table 2, Figure 1), however, they improved more slowly than white students in all three of these areas (Table 3). Students self-identifying as black compared to white was the most important negative predictor in Knowledge, Cognitive Skills, and Overall EL in terms of significance and magnitude (Table 3, Figure 1).

Because we saw pronounced differences in EL levels related to ethnicity (Table 2, Table 3), we also tested interaction terms between ethnicity and time outdoors. Though the full models were minimally impacted, several of the interaction terms between time outdoors and Hispanic ethnicity were significant. Specifically, these interaction terms were significant in the pretest of Affect (Beta = 3.639, p = 0.012) and Behavior (Beta = 4.893, p = 0.016). Additionally, the interaction term between time outdoors and black ethnicity approached significance at the alpha = 0.05 level (Beta = 1.412, p = 0.099) for the Behavior pretest. These findings suggest that time outdoors may impact some components of EL more for Hispanic and black students than for white students. The main effect of time outdoors approached significance (Beta = 0.667, p = 0.058) in the pretest Affect model with the inclusion of the interaction terms, suggesting that although the relationship is stronger among minority students, Affect scores are still positively associated with time outdoors among white students. The time outdoors main effect was not significant in the pretest Behavior scores, suggesting that time outdoors is positively related to Behavior scores among Hispanic students but not significantly among white students. None of the interaction terms were significant for the change in EL models.

### School Attributes

The type of school students attended was related to some areas of EL, supporting hypothesis 5. In the pretest, schools with a higher student/teacher ratio were behind in Knowledge, Title I schools were behind in Behavior, and Charter and Private schools outperformed public schools in Behavior (Table 2). None of these school attributes were significantly related to change in EL over the semester (Table 3). Urban and rural schools performed the same in the pretest, although students in urban schools improved more slowly than those in rural schools over the semester in Cognitive Skills (Table 3). Overall, school attributes had the weakest relationships with EL (Table 3, Figure 1).

## Discussion

### EE and Outdoor Education

The surprising relationship, or lack thereof, between EL and school-wide EE programs (hypothesis 1) may be explained by how EE programs are defined and operationalized. The EE schools were drawn from a list of school-wide EE programs maintained by the NC Office of Environmental Education. Although there are specific qualifications listed on the website, teachers can register their own school based on a personal assessment of their school’s EE program [39]. The low percentage of teachers in EE schools as well as similar percentage of teachers in control schools that stated their students participated in an EE program during the study suggests attendance at an EE school had limited impact on whether a given student was actually exposed to EE programming. Further, teachers may enroll their schools because they perceive deficiencies in EE programming. This may explain why we found a negative association between membership in the EE schools and pretest Affect and Behavior scores. Ultimately, we suspect our findings related to attendance at EE schools have less to do with the efficacy of school-wide EE programs than with how those programs are defined. School-wide EE efforts may in fact be highly effective at building EL and other skills [40], [41], but our results suggest effective evaluations of school-wide EE programs will need to account for what is happening in individual classrooms.

Although there may be some confusion about what constitutes a school-wide EE program, the use of published EE curricula in classrooms is less ambiguous, and our results suggest their use may improve EL. Use of EE curricula was the only variable that was significantly linked to both higher pretest scores and improved Overall EL over the semester. Most of this impact was in the Cognitive Skills component, which is not surprising as guidelines for K-12 EE curricula emphasize ecological knowledge and awareness as well as cognitive skill building [42]. These skills focus on identifying and analyzing complex issues as well as action planning and forming solutions. While many EE programs are content-specific to wetlands (e.g., Project WET), wildlife (e.g., Project WILD), or forests (e.g., Project Learning Tree) [43], the fostering of Cognitive Skills in all of these published EE curricula equip students to engage in and respond to more complex issues including climate change, biodiversity loss, and water quality problems. Studies through the State Education and Environment Roundtable suggest Cognitive Skills built through EL improve academic achievement in reading, writing, math, science, and social studies test scores [41]. Accordingly, use of published EE curricula may be an important tool for improving student achievement in key academic areas beyond EL.

Time spent outdoors complements the use of published EE curriculum by addressing all components of EE other than Cognitive Skills. The relationship with the Affect pretest is not surprising as time outdoors has been linked to improvement in environmental attitudes and intentions [44]. Although time outdoors did not predict a change in Affect, time students spent outside in the fall may have already impacted Affect scores. We did, however, see that time outdoors was associated with improvement in Behavior scores. As improvement in attitudes is generally linked to pro-environmental behavior [45], [46], our data may show that time outdoors fosters pro-environmental attitudes and higher levels of environmental sensitivity which over time leads to more environmentally friendly behavior. Time outdoors also correlated with higher pretest scores in the Knowledge component. Time outdoors can improve student attention in children [47] and is linked to elevated creativity and improved problem solving skills in adults [48], which may explain why contact with nature may improve the more purely academic areas of EL (i.e., Knowledge). Because time outdoors was positively correlated with all areas except Cognitive Skills, use of published EE curriculum and time outdoors together represent a potentially powerful strategy for increasing EL. Our results add to the growing chorus of support for taking kids outside by suggesting outdoor education can promote EL in addition to social [49], mental [50], and physical health [51].

### Teacher Development

Teacher development may be more important than curriculum in terms of predicting EL and may influence EL faster than curriculum. Further, the benefits of teacher development (having a Master’s and 3–5 years of experience) appear to have been largely expressed in the pretest after only one semester of the course. The recent focus on teacher effectiveness in education reform has resulted in numerous studies on how teacher development affects student performance, and the results are mixed. Advanced degrees in education seem to have little or inconsistent effect on overall student performance [52], although subject-specific degrees (e.g. a MS in Biology for science teachers) do seem to have significant impact in Math and Science [53]. As most (75%) of the advanced degrees among the teachers in our study are in education or science education, it appears that our results may conflict with those suggesting training in education does not impact student learning [46]. Our results suggest that a strong background in pedagogical theory and technique is associated with improved student EL.

Interestingly, the relationship between teacher experience and student EL was non-linear. Education literature suggests teacher effectiveness, measured by student achievement, increases rapidly in the first three years of teaching, afterward plateauing for teachers who stay in the profession at least five years [54], [55]. This trend is either attributed to the benefits of on-the-job training provided for early career teachers or the possibility that less-effective teachers leave the profession quickly while more-effective teachers continue teaching past the first few years [56]. Our results add to this research by suggesting that the relationship between teaching experience and student achievement in EL plateaus (e.g., for Cognitive Skills scores in the pretest) after reaching a threshold around 5 years. Specifically, moderately experienced teachers (3–5 years of experience) seem to be more effective at fostering EL, particularly Cognitive Skills, than new teachers, but this effect is not present when considering teachers with more than five years of experience. As this finding parallels research related to teacher experience and other areas of student achievement, perhaps the similar factors of on-the-job training and attrition affect EL. With respect to the pretest of Behavior and change in Affect scores, however, teachers with more than 5 years of experience had students that underperformed those with new teachers. One explanation may be related to loss of idealism and increasing burnout associated with more years of teaching experience [57]. Several studies have suggested inadequate administrative support poses a barrier to inclusion of EE [58] and may contribute to waning enthusiasm and commitment to EE among teachers. Teachers can become increasingly discouraged when not given support to expand use of EE through training or curriculum development [59]. Efforts to promote teacher enthusiasm among veteran EE teachers may be just as important as similar efforts in other disciplines [60], despite the enthusiasm demonstrated by many EE teachers.

### Student Attributes

Our results suggest student attributes have strong relationships with EL, and challenges associated with ethnicity and early education identified in general education research apply to EE efforts as well. Age was negatively related to the change in Behavior and Overall EL, but had no relationship to the pretest in EL, suggesting that while middle school students have similar EL levels among grade levels, older students are slowing down in terms of improvement. This trend is mirrored in research that suggests early education has more bearing on student achievement and even certain measures of life achievement than education efforts with older students [61]. Additionally, older students tend to wane in their interest in science and math in the middle school years [26], which could also explain the slower rate of EL improvement among eighth graders. These findings suggest middle school grades may include an age related tipping point where EE efforts start becoming less effective in promoting EL. We are not arguing that older students cannot benefit from these efforts; rather younger students may have the greatest capacity for learning. Further, the most readily available EE curricula may be more effective for 6^th^ grade students than 8^th^ grade students.

Gender was related to EL in complex ways. Although girls underperformed boys in the pretest for Knowledge, they outperformed them in Affect and Cognitive Skills and improved faster in Knowledge over the course of the semester. The gap in the Knowledge pretest is supported by similar gender trends in science [62]. However, as girls entered the study with more pro-environmental attitudes and higher levels of Cognitive Skills, they were perhaps better positioned for improvement in Knowledge. Girls often underperform boys in the sciences [62], but numerous studies have shown that women and girls hold more positive environmental attitudes and greater levels of concern for the environment [63], [64]. Although overall EL did not differ based on gender, the internal differences associated with Knowledge, Affect, and Cognitive Skills suggest routes to more effective teaching. For example, teachers could use the differing strengths associated with gender to facilitate boys sharing their higher levels of Knowledge with girls while encouraging girls to express their pro-environmental attitudes and utilize their Cognitive Skills to help boys put their Knowledge to use. This strategy could be particularly effective in problem-based group work simulating environmental decision-making.

Ethnicity related differences in EL seem to mirror general education trends; however, the implications may be more complex in EL contexts. Much attention has been paid to achievement gaps between minority students and white students in education literature, and most studies find that individual and school level socio-economic status (SES) are confounded with ethnicity [65]–[67]. These scholars suggest achievement differences are more an issue of poverty than culture. We controlled for school level SES (Title I status), but the differences in EL scores predicted by ethnicity may be at least partially explained by individual SES data. Other explanations for these achievement gaps range from how views of schooling fit into cultural narratives [68] to how teacher expectation bias affects student performance [69].

Although it is possible that achievement gaps in EL are rooted in the same causes as those in other academic areas, differences in cultural perception of the outdoors and access to natural areas may also come into play. Minority groups experience more constraints to natural area access and can be culturally excluded from outdoor recreation [70], [71]. In considering outdoor recreation, safety is of particular concern to some minority groups, including blacks and Hispanics, which may lead to minority children spending less time outdoors than their white counterparts [70]. Additionally, a disproportionately high exposure to environmental risk and decreased availability of natural areas among minority children offers another explanation for the disparities in EL shown in our results [72]. If minority students are exposed to the outdoors less than white students, it would follow that in-school outdoor experiences could have more impact on EL among minority students. Outdoor experiences and contact with nature can be particularly effective in closing gaps in environmental attitudes and awareness associated with ethnicity [73]. The interaction between time outdoors and Hispanic and black students suggest that time outdoors was particularly important in predicting the pretest of Affect and Behavior components among Hispanics and the pretest of Affect among black students. Exposure to nature could help mitigate EL gaps associated with ethnicity, at least among Hispanic and black students. This relationship could be a fruitful area of future study, including the amount, type, or quality of outdoor experience and its impact on EL levels of minority students.

### School Attributes

School characteristics were related to EL in somewhat expected ways. Lower socio-economic status (SES) is generally associated with lower academic achievement [66], but we did not detect this relationship for any dimension of EL except Behavior. Generally, income has been positively associated with environmental behavior [74], [75]. Schools with Title I programs have a higher proportion of lower-income students who, according to these studies, may be less inclined toward environmental concern and behavior. However, more recent literature shows that the link between income and environmental concern is fading and is context dependent [76], [77], which may explain why we found no difference in most EL components associated with SES. In addition to lower SES, bigger class sizes are generally associated with lower academic performance [78], [79]. Our results support this research with a weak negative association between student-teacher ratios and the Knowledge pretest. The relatively low importance of school attributes is encouraging, because curriculum and teacher development are more easily changed than poverty, school types (e.g. charter vs. traditional) or locales.

### Conclusions

Achieving EL through K–12 education is a critical step to creating a public equipped to meet and solve environmental challenges. This study highlights key ways to ensure investments in EE promote EL and ultimately lead to a sustainable future. First, we suggest using published EE curricula and time outdoors in tandem because taken together they foster all four components of EL among middle school students. Published EE curricula including Project WILD, Project WET, and Project Learning Tree were particularly effective at building Cognitive Skills. Future research should address whether other curricular innovations (e.g., service learning) could better engage older students. Time outdoors is one of the only factors that significantly impacts Knowledge, Affect, and Behavior. School-wide EE programs will fail to achieve EL gains unless these programs include tangible changes to curricula in classrooms. Second, despite the informal and volunteer nature of many EE efforts, advanced degrees and years teaching experience are as important in EE as they are in other academic disciplines. EE efforts may improve if advanced degrees among teachers are promoted and teachers are retained for longer periods. The stabilization or even decline in teacher effectiveness after 5 years of experience highlights the need for training, administrative, and structural support for teachers that maintains their enthusiasm and commitment to EE over the long run. Gender based differences in EL appear to complement one another (with each gender excelling in areas where the other does not), suggesting teachers have synergistic opportunities to raise EL levels among both boys and girls. The relationship between ethnicity and EL reveal that although time outdoors may be effective for all students, it may be especially effective for engaging black and Hispanic students.
